# Cyclic Plasticity and Low Cycle Fatigue of an AISI 316L Stainless Steel: Experimental Evaluation of Material Parameters for Durability Design

**DOI:** 10.3390/ma14133588

**Published:** 2021-06-27

**Authors:** Marco Pelegatti, Alex Lanzutti, Enrico Salvati, Jelena Srnec Novak, Francesco De Bona, Denis Benasciutti

**Affiliations:** 1 Department of Engineering, University of Ferrara, via Saragat 1, 44122 Ferrara, Italy; marco.pelegatti@edu.unife.it (M.P.); denis.benasciutti@unife.it (D.B.); 2 Department Polytechnic of Engineering and Architecture, University of Udine, via delle Scienze 208, 33100 Udine, Italy; alex.lanzutti@uniud.it (A.L.); francesco.debona@uniud.it (F.D.B.); 3 Faculty of Engineering & Centre for Micro—and Nanosciences and Technologies, University of Rijeka, Vukovarska 58, 51000 Rijeka, Croatia; jsrnecnovak@riteh.hr

**Keywords:** AISI 316L, low-cycle fatigue, plasticity, hardening, softening

## Abstract

AISI 316L stainless steels are widely employed in applications where durability is crucial. For this reason, an accurate prediction of its behaviour is of paramount importance. In this work, the spotlight is on the cyclic response and low-cycle fatigue performance of this material, at room temperature. Particularly, the first aim of this work is to experimentally test this material and use the results as input to calibrate the parameters involved in a kinematic and isotropic nonlinear plasticity model (Chaboche and Voce). This procedure is conducted through a newly developed calibration procedure to minimise the parameter estimates errors. Experimental data are eventually used also to estimate the strain–life curve, namely the Manson–Coffin curve representing the 50% failure probability and, afterwards, the design strain–life curves (at 5% failure probability) obtained by four statistical methods (i.e., deterministic, “Equivalent Prediction Interval”, univariate tolerance interval, Owen’s tolerance interval for regression). Besides the characterisation of the AISI 316L stainless steel, the statistical methodology presented in this work appears to be an efficient tool for engineers dealing with durability problems as it allows one to select fatigue strength curves at various failure probabilities depending on the sought safety level.

## 1. Introduction

Stainless steels are widely used in different industrial components, ranging from the energy sector to biomedical applications. Within this class of metals, the AISI 316L stainless steel stands out for its distinctive corrosion resistance, combined with good strength, toughness and fatigue properties both at room and medium-high temperatures. Particularly for the latter property, knowledge of the cyclic plasticity behaviour of the material is of great importance, especially in the Low-Cycle Fatigue (LCF) regime.

Several papers can be found in the literature where the cyclic plasticity properties of this type of stainless steel are studied. However, a general mathematical representation of the entire cyclic response of the material up to failure is yet to be found due to the contrasting results found experimentally, especially at high strain amplitudes. For instance, one of the early works on the characterisation of this material showed that, below cyclic plastic strain amplitude of ~0.2%, the material exhibits an initial hardening behaviour followed by the attainment of a maximum stress amplitude and a subsequent mild softening. A third stage may appear depending on the strain amplitude, and it is characterised by a mixed response softening/hardening—sometimes recognised as a plateau when the two contributions are comparable—until failure [1]. Conversely, the same study emphasises that for cyclic plastic strain amplitudes of 0.5% and 1% the hardening regime is not followed by any evident softening before failure. Similar behaviour was observed by other researchers, although they did not report the entire material cyclic response, but only up to 1000 cycles [2]. It is then clear that the cyclic plasticity response of the AISI 316L steel can be separated into three main regimes, in order of appearance: (i) hardening; (ii) softening; (iii) secondary hardening/softening.

Further confirmations on this behaviour can be found in the studies of Pham et al. [3,4], where the third regime (iii) remains essentially of softening type for strain amplitudes ranging from 0.25% to 0.7%, although some evidence of cyclic stabilisation could be detected in some cases.

More recently, Zhou et al. [5] tested two AISI 316L steels showing the same nominal chemical composition and thermal treatment but provided by different suppliers. Similar qualitative cyclic response was obtained, i.e., a third regime (iii) characterised by a hardening/softening behaviour depending upon the applied plastic strain amplitude; transition regime between 0.5% and 0.8%. It is worth mentioning that the two tested samples showed a slightly different initial yield stress (433 MPa vs. 370.4 MPa) in the monotonic tensile stress. Additionally, the material that showed the lowest yield stress manifested an enhanced hardening effect in the third regime (iii). It might therefore mean that the cyclic response is somehow affected by the pre-hardening of the material.

A recent paper authored by Xue-Fang et al. [6] confirmed the transition from softening to hardening behaviour when the strain amplitude is high enough. In particular, in their experiment they found a transition occurring between 0.5% and 0.6%, consistently with what was reported by other authors.

To the best of our knowledge, the micromechanical and physical mechanisms responsible for this peculiar three-regimes cyclic behaviour have not been extensively investigated to cover the whole range of strain amplitude that determines the third regime transition.

In austenitic stainless steels, the secondary hardening is often related to a martensitic transformation induced by the plastic strain, but this behaviour is more common in austenitic stainless steels different from the AISI 316L considered in this work [7,8]. Although in some high-temperature conditions the secondary hardening of AISI 316L may be attributed to the dynamic strain aging [9,10], at room temperature the underlying mechanisms appear to be less intuitive. Of particular interest is the explanation provided by Pham et al. [4] in his experimental and modelling work dealing with strain amplitude up to 0.7%. Under this circumstance (±0.7% strain loading), starting with an as-received material containing a certain amount of dislocation in the form of planar structures, regime (i) is characterised by rapid dislocation densification, particularity in those regions lying next to the grain boundaries. At the beginning of the softening regime (ii), secondary slip systems are activated which allow for a rearrangement of the dislocation to form channel-like structures. Upon further cyclic loading, these channels turn into cellular structures towards the end of the material life. Given that these cellular structures are very effective in combatting the movement of dislocations, the softening behaviour observed regime (ii) may be compensated by a hardening effect, giving rise to regime (iii). Despite the lack of sufficient evidence in Pham’s experimental data, this explanation regarding regime (iii) is very likely to be valid for the other sets of experimental data reviewed in this introductory part of the present paper.

In the case of a mechanical component subjected to loads within the low-cycle regime, the durability analysis is generally performed with the aid of a numerical simulation. It is thus essential to rely on a cyclic plasticity model capable of describing the material behaviour up number of cycles of interest. At that point, the computed strain amplitude, appropriately evaluated through a failure criterion (for example as done by several authors [11,12]), can be employed to estimate the cycles to failure using the Manson–Coffin strain–life curve [13].

These strain–life curves can be obtained through a regression analysis of the experimental data. Such a regression procedure establishes a curve that refers to a 50% failure probability, which may be not sufficiently conservative for a safe durability design. Therefore, depending on the application, a lower failure probability may be sought (e.g., 5% or 1%). Starting from the 50% failure probability curve, a statistical analysis can be performed to obtain the actual so-called ‘design curves’ for any given value of failure probability.

The present study attempts to establish a robust and statistically detailed design approach when dealing with uniaxial low-cycle fatigue of wrought AISI 316L. In order to achieve this goal, low-cycle fatigue tests were performed at several strain amplitude levels to cover the entire case history which has been reported in the literature about the regime (iii). These low-cycle fatigue results were then employed to (1) explore and model the cyclic plasticity response using a Chaboche-based kinematic/isotropic hardening model, suitable to be easily implemented in a commercial FE (finite element) code; (2) evaluate the Manson–Coffin design curves using different techniques and accounting for uncertainties by using statistical methods. Besides providing reliable characterisation of the AISI 316L stainless steel, the present work aims at presenting a statistical approach that may be particularly useful to deal with metals showing such an uncharacteristic cyclic plasticity behaviour. Limitations of the approach are also discussed.

## 2. Plasticity Models: Theoretical Background

One of the goals of this work is to estimate the necessary parameters of cyclic elasto-plastic models to accurately describe the material behaviour, while maintaining its complexity as low as possible. In this section, a brief overview of the models used to accomplish this task is given.

Considering the Von Mises criterion and combined kinematic and isotropic model, the yield surface can be expressed as [14]:

(1) $$\sqrt{\frac{3}{2}\left(\sigma^{\prime }-X\right):\left(\sigma^{\prime }-X\right)}-R-\sigma_{y,0}=0$$

where $\sigma^{\prime }$ is the deviatoric stress tensor and $\sigma_{y,0}$ is the initial yield stress, i.e., the yield stress prior to any loading that plastically deforms the material. In Equation (1), variables X and R represent the effects of kinematic and isotropic hardening, respectively. The variable X is a second-order tensor, called back stress tensor.

The effect of the kinematic hardening is to translate the centre of the yield surface in the stress space while the size of the surface remains constant. The isotropic hardening assumes that, at any stage of loading, the centre of the yield surface remains at the origin and the surface uniformly expand or shrink as plastic strain develops.

As far as the kinematic hardening models are concerned, the most used is perhaps the nonlinear kinematic model of Chaboche, which is represented by the following expressions [14,15,16]:

(2) $$dX=\overset{M}{\underset{i=1}{\sum }}dX_{i}\;;\;dX_{i}=\frac{2}{3}C_{i}d\varepsilon_{pl}-\gamma_{i}X_{i}dp\;\;with\;\;M=1,\;2,\;3,\ldots$$

where each $X_{i}$ component is independent and follows an Armstrong–Frederick model’s type; symbol $d\varepsilon_{pl}$ represent the increment of the plastic strain tensor.

The evolution of a single component $X_{i}$ is governed by a first linear term (known also as the Prager model) and a second nonlinear term. The first linear term represents the strain hardening in which the stress increases proportionally to strain by the hardening modulus $C_{i}$. The second term, on the other hand, is called “recall” term because the nonlinear recovery parameter $\gamma_{i}$ defines the rate at which the hardening modulus starts to decrease as the accumulated plastic strain (p) increases. It is this second term that makes this model nonlinear.

For uniaxial loading, integrating Equation (2) yields [14,15,16]:

(3) $$X=\overset{M}{\underset{i=1}{\sum }}\zeta \frac{C_{i}}{\gamma_{i}}+\left(X_{i,0}-\zeta \frac{C_{i}}{\gamma_{i}}\right)e^{-\zeta \gamma_{i}\left(\varepsilon_{pl}-\varepsilon_{pl,0}\right)}$$

where $\varepsilon_{pl}$ is the plastic strain and coefficient ζ is +1 or −1 during the loading or unloading branch, respectively.

If the loading is monotonic and starts from zero stress and strain condition, the initial state is $X_{i,0}=0$ and $\varepsilon_{pl,0}=0$.

Furthermore, the Chaboche model provides the cyclic stress–strain curve as [14,15,16]:

(4) $$\sigma_{a}=\sigma_{y,s}+\overset{M}{\underset{i=1}{\sum }}\frac{C_{i}}{\gamma_{i}}\tanh\left(\gamma_{i}\varepsilon_{pl,a}\right)$$

where $\sigma_{y,s}$ is the yield stress in the stabilised cycle, $\sigma_{a}$ and $\varepsilon_{pl,a}$ are, respectively, the amplitude of the stress and the plastic strain in the stabilised cycle.

Concerning the isotropic hardening, this is an important effect that needs to be modelled when the materials show a cyclic hardening or softening behaviour. A well-established model used to account for this effect is the nonlinear isotropic hardening model, based on the Voce law [17], governed by the following relations [18]:

(5) $$dR=\overset{Z}{\underset{i=1}{\sum }}dR_{i}\;;\;\;dR_{i}=b_{i}\left(R_{\infty ,i}-R_{i}\right)dp\;\;with\;\;Z=1,\;2,\;3,\ldots$$

where each $R_{i}$ variable is independent, and $R_{\infty ,i}$ and $b_{i}$ are, respectively, the saturated stress and the speed of stabilisation of $R_{i}$. Upon integration, Equation (5) gives:

(6) $$R=\overset{Z}{\underset{i=1}{\sum }}R_{\infty ,i}\left(1-e^{-b_{i}p}\right)$$

In strain-controlled fatigue tests, the plastic strain accumulated after *N* cycles can be approximated as $p≅2\Delta \varepsilon_{pl}N$ where $\Delta \varepsilon_{pl}=2\varepsilon_{pl,a}$ is the plastic strain range per one cycle.

Combining both kinematic and isotropic hardening models, in a uniaxial loading scenario, the stress response becomes:

(7) $$\sigma =\zeta \left(\sigma_{y,0}+R\right)+X$$

where σ is the axial stress.

The outlined models were adopted in this work with the aim of keeping the complexity level of the model as moderate as possible without compromising its accuracy. Overall, this approach considers the kinematic hardening model which controls the shape of a single stress–strain cycle, while the isotropic hardening model regulates the cyclic stress response amplitude.

## 3. Experimental Tests

### 3.1. Material and Testing Setup

The tested material is an AISI 316L stainless steel with chemical composition reported in Table 1. This material was used to manufacture cylindrical specimens, with uniform gauge section of 25 mm in length and 10 mm in diameter, as per [19].

In the present study, after monotonic tensile test characterisation of the material, n.8 low-cycle fatigue tests were conducted at room temperature on an MTS 810 System servo-hydraulic machine with Flex Test SE controller (MTS Systems, Eden Prairie, MN, USA). Each test consisted of a tension-compression cyclic loading under strain control, using a triangular waveform with zero mean strain. An extensometer (MTS 634 model), with a gauge length of 25 mm and a +5 mm/−2.5 mm range of measure, was used to record and control the axial strain. A force transducer with a capacity of 100 kN was used to monitor the axial force during cyclic testing. The system, in strain control, was tuned before the tests. The loading frequency was adjusted for each sample in order to keep a constant strain rate of 4 × 10^−3^ s^−1^. The experiments were stopped before the complete separation of the specimen when the strain exceeded the safety limit imposed to the tensile machine.

### 3.2. Brief Analysis of the Experimental Material Behaviour

**Figure 1:**
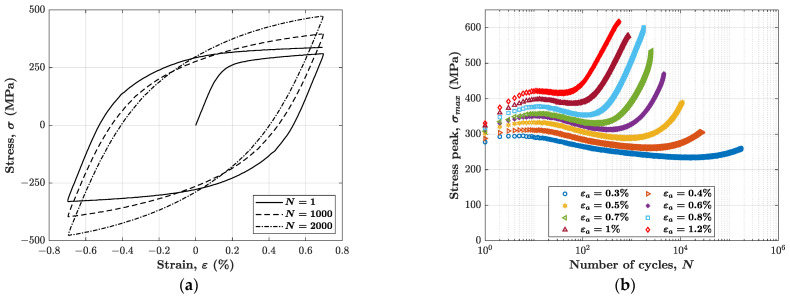
Experimental behaviour observed: (**a**) example of three stress-strain cycles in the test with 0.7% strain amplitude; (**b**) cyclic stress response in all tests carried out with different strain amplitudes.

The cyclic stress–strain response observed in the experiment at $\varepsilon_{a}$ = 0.7% is reported in Figure 1a. For more clarity, the figure only plots the 1st, 1000th and 2000th cycle.

The cyclic stress response is the variation of the stress amplitude as a function of the increasing number of cycles during the test. Since the experimental findings showed that the axial stress in every cycle is nearly symmetric (see Figure 1a), the variation of stress amplitude was obtained by simply monitoring the variation of the tensile peak stress, see Figure 1b. As expected, this material shows the characteristic three regimes discussed earlier, i.e., hardening (i), softening (ii) and mixed secondary hardening/softening (iii). For our specific case, regime (iii) is characterised by a distinctive secondary hardening behaviour, even for relatively low strain amplitudes, which is in contrast with what has been reported in the literature [5,6]. Nonetheless, as the applied strain amplitude becomes higher and higher, the secondary hardening effect becomes more and more enhanced. The distinctive outcome of this experimental campaign is, in fact, that the stress peak reaches values as high as 600 MPa in the regime (iii) at applied strain amplitudes between 0.8% and 1.2%. A similar trend has been experienced by Zhou et al. [5] in only one instance for strain amplitudes of 1.25%, although not reaching such a high stress peak value.

Curiously, the regime (iii) presents a linear increase of the tensile stress peak.

**Figure 2:**
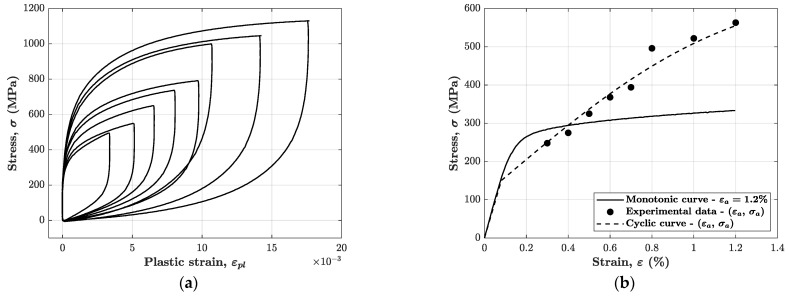
Masing behaviour analysis results: (**a**) stress–plastic strain hysteresis cycles rigidly translated to the origin (0,0); (**b**) comparison between the monotonic curve and the cyclic curve passing through the experimental points $\left(\varepsilon_{a},\sigma_{a}\right)$.

With the objective of establishing a reliable predictive durability model, it could be helpful to evaluate the Masing behaviour of the material, based on the obtained experimental data. As Figure 2a shows, the plastic strain for all the strain amplitudes was firstly calculated. The stress versus plastic strain loops obtained in this way are translated to the origin, in such a way that all the points corresponding to the lowest stress magnitudes overlap with each other. It is evident that in the case of AISI 316L the material displays a non-Masing behaviour given that the tensile branches of the “stabilised” hysteresis loop do not overlap. Moreover, in a material with Masing behaviour the cyclic curve is obtained by simply translating the monotonic curve upward or downward, which clearly is not the case for the AISI 316L, see Figure 2b.

## 4. Plasticity Models: Identification of Material Parameters

### 4.1. Young’s Modulus and Yield Stress

Young’s modulus and yield stress are the first parameters that are estimated from the experimental data. Young’s modulus E was identified from the initial tensile loading part of each test, and also from the tensile and compressive branches corresponding to half of the number of cycles to failure. These evaluated values did not show any significant deviation, therefore, all the estimated values were averaged out to give a single value of 191626 MPa.

Initial yield stress $\sigma_{y,0}$ was identified from the first loading portion at the beginning of the test. Since the material does not exhibit a discontinuous yielding (i.e., a clear yield point), the initial yield stress was determined conventionally by considering an offset in plastic strain. If the usual offset of 0.2% is considered, a yield stress of 310 MPa is estimated from monotonic tensile test. Nevertheless, for cyclic plasticity, the value of 0.2% turned out to be not the best choice to capture the materials behaviour in the model calibration that follows [20]. Instead, a plastic strain offset value of 0.0025% was considered in our study, as suggested by [5]. The initial yield stress was estimated from each test, showing a negligible scatter. An average value of 169 MPa was calculated.

For the identification of hardening model parameters, it is also important to estimate the yield stress in the tensile and compressive branches of the “stabilised” cycle of each test. In this case the value of the plastic strain offset is set to 0.01%. The reason why this value is bigger than the offset chosen for the initial yield stress is that the acquisition resolution is far better in the first loading portion of the very first cycle, than in the following ones; the choice of 0.01% as a plastic strain offset did not lead to significant errors in the assessment. For the following calibration steps, an average value, $\sigma_{y,s}$, of the two yield stresses for tension and compression branches will be used for each cycle, the average value being different for various strain amplitudes.

### 4.2. Kinematic Hardening Model

After having identified the Young’s modulus and the yield stress, it is possible to estimate the parameters of hardening models. The procedure consists firstly of the estimation of the Chaboche kinematic model parameters, followed by the calibration of the nonlinear isotropic model parameters.

The kinematic model parameters are usually identified through curve fitting [21]. In particular, it is necessary to calculate the amplitude of the back stress $X_{a}=\sigma_{a}-\sigma_{y,s}$ and the amplitude of plastic strain $\varepsilon_{pl,a}$ in the stabilised cycles of each test at different strain amplitude $\varepsilon_{a}$. The experimental points obtained by the procedure described so far are then interpolated by the expression in Equation (4) in order to obtain $C_{i}$ and $\gamma_{i}$. The curve found in this way is called the ‘back stress cyclic curve’.

Applying this procedure is however not straightforward, since the AISI 316L material never stabilises. A possible strategy could be that of considering the cycles at half the number of cycles to failure, $N_{f}/2$. Such an approach is not suitable in this case, as $N_{f}/2$ occurs on the secondary hardening stage (see Figure 1b), where cycles present a different shape with respect to those of the first part of the test. Furthermore, this last stage of cyclic stress response, i.e., secondary hardening, is highly dependent on strain amplitude.

To overcome the upper mentioned problems, an alternative approach was adopted.

Considering that, during the secondary hardening phase, the shape of the cycles changes considerably, the calibration of the kinematic parameters was performed on the cycles at the end of the softening stage. Since in the three tests with the higher strain amplitude (0.8%, 1%, 1.2%) the secondary hardening is dominant, only tests with a strain amplitude from 0.3% to 0.7% were considered in the improved fitting procedure that follows.

To start with, it was decided to fit the tensile branch of the cycle tested at the highest strain amplitude 0.7%, as suggested in some studies [22]. For this strain amplitude, the end of the softening stage occurs after 200 cycles. This choice provided preliminary guess parameter values for the kinematic hardening model.

**Figure 3:**
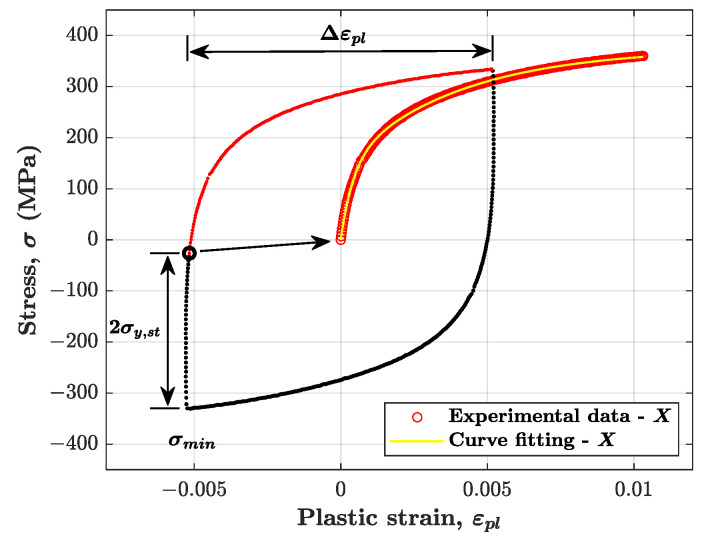
Curve fitting to find kinematic hardening parameters applied to the tensile branch of the 200th cycle of the test at 0.7% strain amplitude.

The fitting procedure was applied to the following expression:

(8) $$\sigma -\sigma_{min}-2\sigma_{y,st}≅X=2\overset{M}{\underset{i=1}{\sum }}\frac{C_{i}}{\gamma_{i}}\frac{1-e^{-\gamma_{i}\varepsilon_{pl}}}{1+e^{-\gamma_{i}\Delta \varepsilon_{pl}}}\;\;for\;\;\left(\sigma -\sigma_{min}-2\sigma_{y,st}\right)\geq 0$$

where $\sigma_{min}$, $\sigma_{y,st}$ and $\Delta \varepsilon_{pl}$ are evaluated from the experimental cycle (see Figure 3); σ and $\varepsilon_{pl}$ are the set of values describing the branch of the cycle to be fitted, whereas $C_{i}$ and $\gamma_{i}$ are the fitting parameters to be determined. The proposed fitting procedure is slightly approximated as it neglects the influence of isotropic hardening in one cycle.

The right hand side of Equation (8) follows from translating Equation (3) to the origin, which is obtained by taking $\varepsilon_{pl,0}=0$ in the exponent and by adding the sum of M terms $X_{i,0}=-\frac{C_{i}}{\gamma_{i}}\tanh\left(\gamma_{i}\varepsilon_{pl,a}\right)$ to the left of the equal sign in Equation (3). Figure 3 displays the fitting result for the experimental 200th cycle of the test at 0.7% strain amplitude. As can be seen, the portion of the tensile branch of the cycle was translated to the origin and then used for the curve fitting.

**Figure 4:**
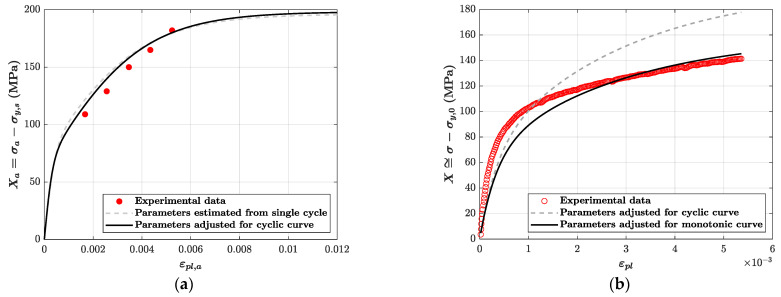
Kinematic hardening parameter (X) evaluation: (**a**) from cyclic curve; (**b**) from monotonic curve.

The parameters estimated with M=2 are $C_{1}=172641\;\mathrm{MPa}$, $\gamma_{1}=2358$, $C_{2}=30855\;\mathrm{MPa}$ and $\gamma_{2}=251$. These parameters were inserted in Equation (4) to compute the back stress cyclic curve (see dashed line in Figure 4a), which is then compared to the experimental points ($X_{a}\;,\;\varepsilon_{pl,a}$) (see markers in Figure 4a). The experimental points are derived from the cycle corresponding to the end of the softening phase in each of the five tests with strain amplitude from 0.3% to 0.7%. Eventually, an additional parameter refinement was carried out to improve the fit (see continuous line in Figure 4a): $C_{1}=189500\;\mathrm{MPa}$, $\gamma_{1}=2950$, $C_{2}=33500\;\mathrm{MPa}$ and $\gamma_{2}^{*}=250$.

A comparison of simulated and experimental monotonic curves was also made. The monotonic curve was simulated with only the kinematic hardening model, once again by neglecting the contribution of the isotropic hardening. Actually, the comparison in Figure 4b considers the monotonic curve shifted downward by $\sigma_{y,0}$, so that they refer to $X≅\sigma -\sigma_{y,0}$. A fitting improvement (consider that the dashed line in Figure 4b is obtained by using the parameters calibrated from the cyclic curve at the end of the softening stage) can be achieved with a slight correction of the $\gamma_{2}^{*}$ parameter of the kinematic model, which becomes $\gamma_{2}=350$. The continuous line shown in Figure 4b, which provides a better fitting, is thus obtained. These last parameters are those used in the final comparison between experimental and simulated data, see Table 2.

### 4.3. Isotropic Hardening Model

Isotropic model parameters were calibrated by considering the experimental response up to the end of the softening stage (i.e., the local minimum of the cyclic response curve), consistently with the choice followed in the case of the kinematic model.

Since the material shows a sequence of hardening followed by softening stage, it was decided to use two terms (Z=2) of the isotropic hardening model in Equation (5).

In addition, the observed material hardening or softening phenomena depend upon the strain amplitude, so that the values of $R_{\infty ,i}$ are different for each test. Therefore, it was necessary to find different parameters for each test.

A further moderate improvement in the estimates of isotropic parameters can be achieved if the kinematic contribution is removed from the experimental cyclic stress response. For this purpose, the kinematic hardening model calibrated before is used.

**Figure 5:**
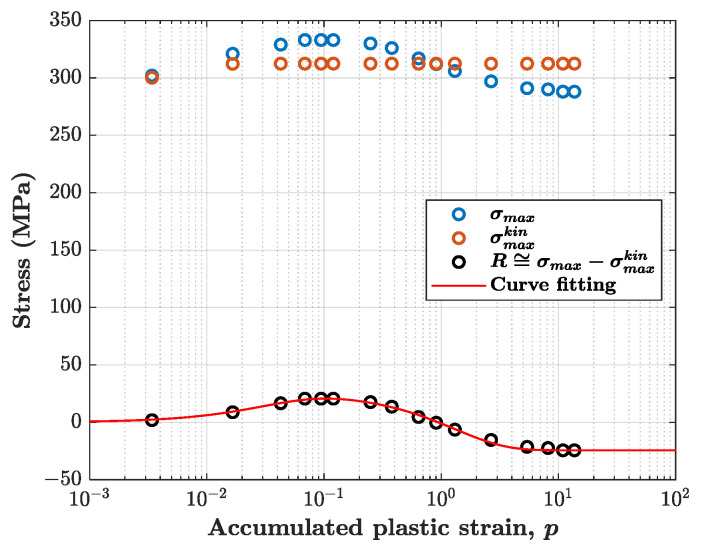
Example of curve fitting of Equation (9) to find isotropic hardening parameters for the test with 0.5% strain amplitude.

The expression to be fitted is:

(9) $$\sigma_{max}-\sigma_{max}^{kin}≅R=R_{\infty ,1}\left(1-e^{-b_{1}p}\right)+R_{\infty ,2}\left(1-e^{-b_{2}p}\right)$$

where $\sigma_{max}$ are the experimental stress peaks, $\sigma_{max}^{kin}$ are the stress peaks obtained considering only the kinematic model, and p is the accumulated plastic strain evaluated experimentally; $R_{\infty ,i}$ and $b_{i}$ are the fitting parameters to be determined. In the curve fitting, the sum of the two saturated values must be equal to $R_{\infty ,1}+R_{\infty ,2}=\sigma_{max,s}-\sigma_{max,s}^{kin}$, where the subscript s indicates that the stress peak is referred to the cycle at the end of the softening stage. Figure 5 shows how the method applies to a single test. Open orange markers represent the stress peaks obtained by simulating the cyclic response with only the contribution of the kinematic model, which saturates almost immediately and thus gives constant stress peaks after few cycles. The difference between the open blue marker values, which are the experimental stress peaks, and the open orange marker values was attributed to the isotropic hardening model. Table 2 lists all the estimated parameters.

To make the material model employable also for strain ranges different from those tested experimentally, it was decided to interpolate the evaluated parameter values with a 2nd order polynomial function of the strain amplitude. For example, the parameter $R_{\infty ,1}$ for a particular strain amplitude $\varepsilon_{a}$ can be found using the following polynomial function:

(10) $$R_{\infty ,1}=A_{1}+A_{2}\;\varepsilon_{a}+A_{3}\;\varepsilon_{a}^{2}$$

**Figure 6:**
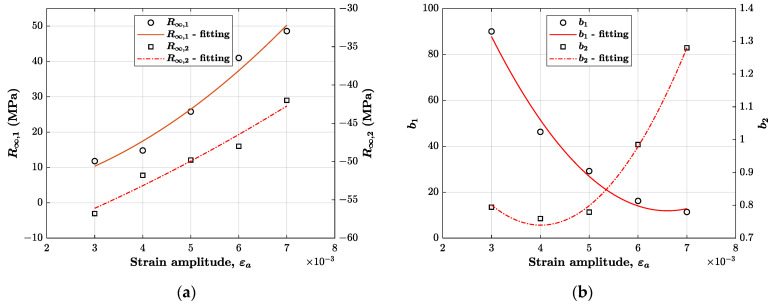
Curve fitting for each isotropic model parameter using a 2nd order polynomial function of the strain amplitude: (**a**) saturated stress $R_{\infty ,i}$; (**b**) speed of stabilisation $b_{i}$.

The outcome of the fitting procedure can be visualised in Figure 6. From this figure, it is clear that 2nd order polynomial functions are suitable for this purpose and produce a negligible error. The estimated coefficients of the functions ($A_{1},\;A_{2}$ and $A_{3}$) for each parameter are reported in Table 3.

It is important to remark that these functions could also be used to find estimated model parameters lying outside the experimentally tested range, although the same accuracy would no longer be guaranteed, particularly for strain ranges far from the tested strain amplitude limits.

### 4.4. Model vs. Experiment Comparison

This section compares experiment with simulation. The simulation was carried out by the algorithm described in Appendix A, which allows one to simulate the uniaxial response. In this simulation, the parameters taken into account are reported in Table 2. Each strain amplitude is characterised by different isotropic hardening parameters. Furthermore, for every simulation the values of Young’s modulus and initial yield strength are respectively E=191626 MPa and $\sigma_{y,0}=169\;\mathrm{MPa}$.

**Figure 7:**
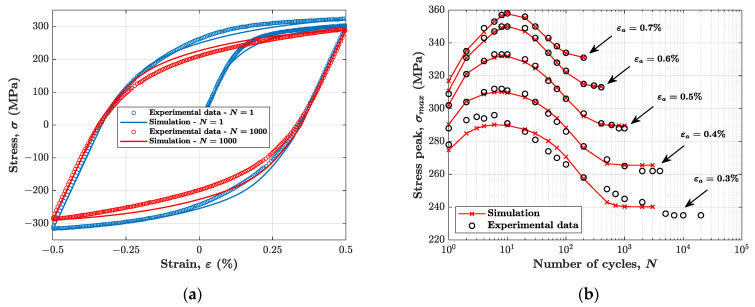
Comparison between experimental data and simulation with kinematic hardening parameters calibrated on monotonic curve and isotropic hardening parameters different for each test: (**a**) stress–strain cycles for test with 0.5% strain amplitude; (**b**) cyclic stress response.

Figure 7 compares the experimental data with the results from simulations with both the kinematic and the isotropic hardening models. Figure 7a compares two stress–strain cycles (1st and 1000th) for a strain amplitude of 0.5%, whereas Figure 7b shows the evolution of the peak stress in each cycle, throughout each test. The comparison shows how the cyclic stress response is simulated with more than satisfactory accuracy. As a measure of the model accuracy, the mean percentage absolute error (MAPE) of the stress peaks for each evaluated test (i.e., for a fixed strain amplitude) was assessed; the results are reported in Table 4. MAPE is an error index defined as follows:

(11) $$\mathrm{MAPE}=\frac{100}{l}\overset{l}{\underset{i=1}{\sum }}\left|\frac{\sigma_{max,i}^{exp}-\sigma_{max,i}^{sim}}{\sigma_{max,i}^{exp}}\right|$$

where l is the number of stress peaks taken into consideration to calculate the index for a single test, $\sigma_{max,i}^{exp}$ is the i-th experimental stress peak and $\sigma_{max,i}^{sim}$ is the i-th simulated stress peak. The present error analysis was carried out considering a total of n. 12 peak values. In addition to MAPE index, in Table 4 is shown the absolute percentage error (APE) of the last stress peak simulated (i.e., at the end of the softening stage) for each strain amplitude considered.

It can be noted, from Figure 7a, that the compressive branch of the stress–strain cycles is marginally overestimated in terms of absolute stress value.

## 5. Low-Cycle Fatigue Curves

The results of low-cycle fatigue tests were also used for estimating the strain–life (Manson–Coffin) equation, which relates the total strain amplitude $\varepsilon_{a}$ to the number of reversals to failure $2N_{f}$:

(12) $$\varepsilon_{a}=\varepsilon_{el,a}+\varepsilon_{pl,a}=\frac{{\sigma_{f}}^{\prime }}{E}{\left(2N_{f}\right)}^{b^{\prime }}+{\varepsilon_{f}}^{\prime }{\left(2N_{f}\right)}^{c^{\prime }}$$

where $\varepsilon_{el,a}$ and $\varepsilon_{pl,a}$ are the elastic and plastic strain amplitudes, respectively. Other symbols are fatigue strength coefficient $\sigma_{f}^{\prime }$, fatigue strength exponent $b^{\prime }$, fatigue ductility coefficient $\varepsilon_{f}^{\prime }$, fatigue ductility exponent $c^{\prime }$, elastic modulus E.

In a log-log diagram, Equation (12) is the sum of two straight lines. Therefore, the equation parameters can be estimated by a linear regression analysis of experimental data, which must be carried out separately for the elastic strain and the plastic strain parts. The linear regression model is y=A+Bx+δ, where $y=log\left(2N_{f}\right)$ and $x=log\left(\varepsilon_{a}\right)$ denote, respectively, the log-transformed fatigue life and strain amplitude, where obviously $\varepsilon_{a}=\varepsilon_{el,a}$ or $\varepsilon_{a}=\varepsilon_{pl,a}$ depending on whether the elastic or plastic strain amplitude is considered. In the regression model, the quantity δ~N(0,s) represents a normally distributed random variable with zero mean and constant standard deviation s (where constant means “not a function of x”; this type of model is called homoscedastic). The random variable δ allows the regression model to account for the inherent (aleatory) variability of fatigue life $2N_{f}$.

According to the regression model introduced so far, at each strain amplitude *x* the fatigue life *y* is normally distributed with mean A+Bx and standard deviation s. The model parameters are not known in advance, but they must be estimated from a set of n experimental pairs ($\varepsilon_{a,i}$, $2N_{f,i}$), i=1,…n. After separating the total strain amplitude into elastic and plastic part, one obtains n pairs of log-transformed variables $y_{i}=\log\left(2N_{f,i}\right)$, $x_{el,i}=log\left(\varepsilon_{el,a,i}\right)$ or $x_{pl,i}=log\left(\varepsilon_{pl,a,i}\right)$, which are input in standard formulae for regression analysis [23].

The formulae yield the values of the estimators $\hat{A}$, $\hat{B}$, $\hat{s}$, and therefore the “median” strain–life $\hat{y}=\log\left(2{\hat{N}}_{f}\right)=\hat{A}+\hat{B}x$. The ‘cap’ specifies that the estimators are characterised by a statistical (epistemic) uncertainty that comes from using a limited number of experimental data (this uncertainty would vanish if n were infinite).

It is possible to retrieve the parameters of the “median” strain–life curve by an inverse log-transformation of the regression estimators:

(13) $$\frac{{\hat{\sigma }}_{f}^{\prime }}{E}=10^{\left(-{\hat{A}}_{el}/{\hat{B}}_{el}\right)};\hat{b^{\prime }}=\frac{1}{{\hat{B}}_{el}};{\hat{\varepsilon }}_{f}^{\prime }=10^{\left(-{\hat{A}}_{pl}/{\hat{B}}_{pl}\right)};\hat{c^{\prime }}=\frac{1}{{\hat{B}}_{pl}}$$

where subscripts “el” and “pl” stand for elastic and plastic. The strain–life curve can be promptly found by calculating the total strain amplitude as the sum of elastic and plastic strain components, see Equation (12). The estimated parameters are listed in the first row of Table 5.

### 5.1. Approximate Strain–Life Curves from Monotonic Tensile Properties

Since low-cycle fatigue tests are costly and time-consuming, different methods have been proposed to approximate the strain–life curve parameters from monotonic tensile properties or even hardness measurements [24,25]. This type of approximated approach is proven to be particularly useful in the early design phase, when only a rough estimate of the strain–life curve is sought.

Among such type of approximations, a noteworthy example is the “Universal Slopes Equation” (USE) proposed by Manson [26,27]:

(14) $$\Delta \varepsilon =\Delta \varepsilon_{el}+\Delta \varepsilon_{pl}=3.5\;\left(\frac{\sigma_{\mathrm{uts}}}{E}\right)N_{f}{}^{-0.12}+D^{0.6}N_{f}{}^{-0.6}$$

where $\sigma_{uts}$ is the tensile strength and D=ln[100/(100−Z)] the ductility, which depends on the percent necking Z%. The two exponents −0.12 and −0.6 are assumed to be equally valid for all types of materials. The model was calibrated on low-cycle fatigue data for ferrous and non-ferrous alloys (e.g., steel, silver, magnesium, titanium, aluminium). Like as the regression curve, also the USE in Equation (14) represents a “median” curve that refers to a probability of failure of 50%.

Note that the above equation refers to strain ranges, i.e., twice as the strain amplitude. It is convenient to rewrite it in a form similar to the Manson–Coffin equation:

(15) $$\varepsilon_{a}={\left(\frac{\sigma_{f}^{\prime }}{E}\right)}_{USE}{\left(2N_{f}\right)}^{-0.12}+{\left(\varepsilon_{f}^{\prime }\right)}_{USE}{\left(2N_{f}\right)}^{-0.6}$$

where:

(16) $${\left(\frac{\sigma_{f}^{\prime }}{E}\right)}_{USE}=\frac{3.5}{2^{\left(1-0.12\right)}}\left(\frac{\sigma_{uts}}{E}\right);{\left(\varepsilon_{f}^{\prime }\right)}_{USE}=\frac{D^{0.6}}{2^{\left(1-0.6\right)}}$$

The USE model assumes that, in the strain–life curve, the elastic part is directly proportional to the material strength, whereas the plastic part is proportional to the material ductility. As a result, the USE model predicts that, at higher strains, the fatigue life mainly depends on the material ductility and, at lower strains, on the static strength, as it is usually the case in high-cycle fatigue.

**Figure 8:**
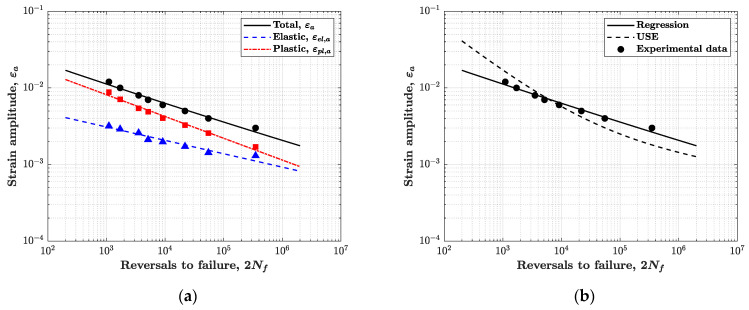
(**a**) Manson–Coffin strain–life curve from regression analysis, with contributions of elastic, plastic and total strain amplitude; (**b**) comparison between the Manson–Coffin curve and the Universal Slopes Equation model.

For the AISI 316L steel, the values E=194699 MPa, $\sigma_{uts}=651\;\mathrm{MPa}$ and D=1.347 were estimated from tensile test (note: the value of E estimated from the tensile test is marginally different from the average value of E obtained from all fatigue tests, see Section 4.1). The Manson–Coffin parameters from USE approximation are listed in the second row of Table 5. As shown in Figure 8b, the agreement between the USE and Manson–Coffin curves is not particularly satisfactory; the USE curve is steeper and has different intercepts for the elastic and plastic parts. On the other hand, this result has somehow to be expected, given the degree of approximation of the USE model.

### 5.2. Statistical Methods and Strain–Life Design Curves

The strain–life relationship obtained by regression, $\hat{y}=\hat{A}+\hat{B}x$, connects all the mean values $\hat{y}$ for each x, and therefore is a “median” curve that is defined for a failure probability α=50%, not sufficiently conservative for safe durability design. A much lower probability must be specified; this corresponds to defining a so-called design (or characteristic) strain–life curve, shifted to the left of the “median” curve as:

(17) $${\hat{y}}_{d}=\hat{y}-K\left(\alpha ,\beta ,n,x,x\right)\cdot \hat{s}$$

where $\hat{y}=\hat{A}+\hat{B}x$ is the “median” curve by regression (for α = 50%) that links the (log-) reversals to failure $\hat{y}=log(2{\hat{N}}_{f})$ to the (log-) strain amplitude $x=log\left(\varepsilon_{a}\right)$, either elastic or plastic. Symbol $\hat{s}$ is the standard deviation estimated by regression analysis. The equation has to be applied separately for the elastic and plastic parts in the strain–life equation. The obtained ‘elastic’ and ‘plastic’ design curves have to be summed to find the design curve in terms of total strain amplitude.

The form of the previous equation emphasises that, in the most general case, the statistical factor K(α,β,n,x,x) is a function of the prescribed failure probability α and confidence β (if required), on the number n and specific amplitudes $x=\left(x_{1},\;x_{2},\;\ldots ,\;x_{n}\right)$ used in fatigue tests, and on the strain amplitude $x=log\left(\varepsilon_{a}\right)$ at which the design fatigue life ${\hat{y}}_{d}=log\left(2N_{f,d}\right)$ is being computed. When the experimental tests are performed, the two parameters n and x take on fixed values. For this reason, to simplify the notation, the statistical factor will be written simply as K(α,β,x).

Various methods are available in the literature to define the value of K(α,β,n) and thus the design fatigue life ${\hat{y}}_{d}$. In the following, the methods are subdivided into two categories as (i) deterministic method (also called “2 sigma” or “3 sigma”) and (ii) statistical methods, which further includes three approaches (confidence interval, tolerance interval, prediction interval).

**Figure 9:**
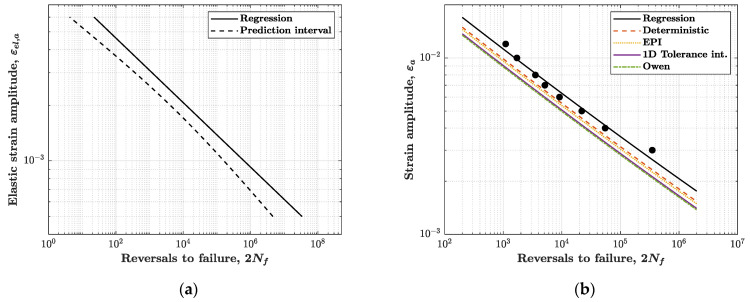
(**a**) Example of “hyperbolic” design curve obtained with the prediction interval method for the elastic strain amplitude; (**b**) comparison between Manson–Coffin and design curves from different methods.

When coefficient K(α,β,x) is a function also of the strain amplitude x, the design curve is no longer straight, but rather takes a hyperbolic-like shape—an example is depicted in Figure 9a. Obtaining such hyperbolic curves require statistical methods that could not be known to non-specialists, or that simply are impractical to use. For this reason, a straight-line approximation (with K(−) = const. and independent of x) is often preferred, since it only gives a negligible loss in confidence compared to the exact solution. In this case, the design curve becomes straight and simply shifted to the left of the “mean” curve as:

(18) $${\hat{y}}_{d}=\left(\hat{A}-K\left(\alpha ,\beta \right)\cdot \hat{s}\right)+\hat{B}x$$

In which $\hat{A}-K\left(\alpha ,\beta \right)\cdot \hat{s}$ is a new constant that only depends on failure probability α (the survival probability is 1−α) and confidence β. Though approximated, these methods undoubtedly have the advantage to permit the parameters $\sigma_{f}^{\prime }$, $\varepsilon_{f}^{\prime }$ of the ‘design’ Manson–Coffin curve to be expressed in closed-form as:

(19) $${\left(\frac{{\hat{\sigma }}^{\prime }{}_{f}}{E}\right)}_{d}=10^{\left[-\left({\hat{A}}_{\mathrm{el}}-K\cdot \hat{s}\right)/{\hat{B}}_{\mathrm{el}}\right]}\;\;\;\;\;\;\;\;\;\;\;{\left(\hat{\varepsilon }\prime_{f}\right)}_{d}=10^{\left[-\left({\hat{A}}_{\mathrm{pl}}-K\cdot \hat{s}\right)/{\hat{B}}_{\mathrm{pl}}\right]}$$

Note that, instead, the exponents remain unchanged and equal to the values $\hat{b\prime }$, $\hat{c\prime }$ obtained by regression analysis (in other words, the elastic and plastic design curves are straight lines translated from the regression lines).

The following paragraphs will survey several methods for estimating the design curves, with particular focus on approximate methods that provide a constant value of K(−) and thus prove to be particularly useful in practical situations.

As a final remark, while statistical methods are routinely applied in the field of high-cycle fatigue to define a design curve, their use in low-cycle fatigue seems to be less frequent, although some studies have pointed out its importance [28,29]. The results presented in the following aim to highlight that design curves play an important role in low-cycle fatigue, too.

#### 5.2.1. Deterministic Method (“2 sigma” or “3 sigma”)

This approach neglects the statistical (epistemic) uncertainty of regression estimators and it postulates that $\hat{A}=A$, $\hat{B}=B$ and $\hat{s}=s$, that is, the estimators coincide with the “true” parameters that would be obtained with n infinite. In this method the (log-)fatigue life y is a normal variable with mean $\hat{y}$ and standard deviation $\hat{s}$. Accordingly, for a given survival probability 1−α, the statistical factor is $K_{det}=z_{1-\alpha }=\Phi^{-1}\left(1-\alpha \right)$, where Φ(z) is the cumulative distribution of the standardised normal variable z. For example, for a failure probability α=1%, it is $K_{det}=z_{0.99}=2.3263$. Though certainly simple, this method is not conservative, as it neglects the statistical variability of $\hat{A}$, $\hat{B}$ and $\hat{s}$.

#### 5.2.2. One-Side Tolerance Interval Method

A tolerance interval establishes a region that encloses a given fraction of the population of a random variable. It is trivial to compute a tolerance interval for a normal random variable for which its mean value μ and standard deviation s are known [30]. From probability theory, for example, it is known that 95% of the values of a normal distribution fall within the two-side interval μ ±1.96 s, where $z_{0.975}=\Phi^{-1}\left(0.975\right)=1.96$ [30]. If, instead, mean and standard deviation are only known through their estimates $\hat{\mu }$ and $\hat{s}$, and additional source of uncertainty is present.

With reference to the Gaussian variable $y=log\left(2N_{f}\right)$ at given x, the value ${\hat{y}}_{d}$ in the design curve Equation (17) corresponds exactly to the definition of a one-side interval $y\leq {\hat{y}}_{d}$ enclosing a percentage α of the values of y. Additionally, in this case, for variable y the regression analysis only yields the estimators of the mean $\hat{A}+\hat{B}x$ and standard deviation $\hat{s}$, which thus have an additional uncertainty not present in the “true” values. The statistical variability of the estimators does not allow the statistical factor K(−) to be computed through the cumulative distribution Φ(z), as in the “deterministic method”.

It is necessary to evaluate K(−) by following a different approach able to account for the confidence β of the estimators. Specifically, the approach seeks the value K(α,β,n) by which to identify the bound ${\hat{y}}_{d}$ of the tolerance interval $y\leq {\hat{y}}_{d}$, so that the interval contains (with a given confidence β) a percentage α of the values of y. Factor K(α,β,n) becomes a function of the failure probability α, of the confidence β and of the size n of the statistical sample. For a normal distribution, the values of K(α,β,n) are tabulated [31,32]. For example, for the values n=7, α=1%, β=90%, it is K(α,β,n)=3.9720.

The design line ${\hat{y}}_{d}=\hat{y}-K\left(\alpha ,\;\beta ,n\right)\cdot \hat{s}$ obtained by this method ensures that, in the long run, 100β% of times, the failures at $y\leq {\hat{y}}_{d}$ will occur with probability α.

The method of the tolerance interval yields a constant K(α, β,n) across the entire interval of x, which corresponds to a straight design line. The method is, however, correct only for a single random variable. In the regression case (having two variables x and y), the method turns out to be approximate, since it neglects the statistical uncertainty of regression estimators [33].

When the method of tolerance interval is applied to the linear regression (“Owen’s method” [34]), the factor K(α, β,n,x) becomes also a function of the strain level x and, accordingly, the design curve is no longer straight. The application of this approach, however, presents some practical difficulties. A possible approximation (“approximate Owen’s method”) considers an approximated value $K_{app}\left(\alpha ,\;\beta ,n\right)$ that is constant on the whole range of x. By doing this, the design curve becomes again a straight line ${\hat{y}}_{d}=\hat{y}-K_{app}\left(\alpha ,\;\beta ,n\right)\cdot \hat{s}$.

The theoretical details of this method—here omitted as being of no much interest—can be found in [29]. The main advantage of this method is that the values of $K_{app}\left(\alpha ,\;\beta ,n\right)$ are already tabulated in [32] for different values of n, α and β (note that some values in [29] are not correct). For example, for given n=7, α=1%, β=90%, it is $K_{app}\left(\alpha ,\;\beta ,n\right)=4.3187$.

#### 5.2.3. Prediction Interval Method

A prediction interval is a region in which a future value of a random variable will fall with a given probability. When calculating this interval, the uncertainty associated to the future random observation must be added to the uncertainty of estimators $\hat{A}$, $\hat{B}$ and $\hat{s}$.

The expression of the prediction interval (see [35]) shows that K(α, n,x) is a “t-Student” random variable, and it depends on the failure probability α, strain amplitude x and sample size n, but not on the confidence β. Accordingly, the design curve is not straight (see an example in Figure 9a), which makes computation somehow more complicated. For this reason, an approximate method (called “equivalent prediction interval”, EPI) was proposed in [35] with the purpose to compute a constant factor $K_{EPI}\left(\alpha ,\;n\right)$ by which to replace K(α, n,x).

The idea of the EPI method is to assume that the normal variable y has a constant standard deviation $\sigma_{0}=\hat{s}\cdot g\left(\alpha ,n\right)$ obtained by means of a correction factor g(α,n) that quantifies the uncertainty of the estimators $\hat{A}$, $\hat{B}$, $\hat{s}$. In [35], the following expressions (valid for 6≤n≤50, 0.01≤α≤0.15) were proposed:

(20) $$g\left(\alpha ,n\right)=\exp\left[\Lambda \left(\alpha \right){\left\{\ln n\right\}}^{-\Psi \left(\alpha \right)}\right]\;\Lambda \left(\alpha \right)=1.56{\left[\tan h^{-1}(1-\alpha )\right]}^{1.12}\;\;;\;\;\Psi \left(\alpha \right)=3.32-1.7\alpha$$

After having determined $\sigma_{0}$, the design curve turns out to be ${\hat{y}}_{EPI}=\hat{y}-\left[K_{det}\cdot g\left(\alpha ,n\right)\right]\cdot \hat{s}$, where $K_{det}$ is the factor from the deterministic method (see Section 5.2.1). By analogy with other methods described so far, it is also possible to define $K_{EPI}=K_{det}\cdot g\left(\alpha ,n\right)$. For example, for n=7, α=1%, it is $K_{EPI}=3.8924$.

#### 5.2.4. Results

The statistical methods described in the previous sections were applied to compute the design strain–life curves of the AISI 316L steel. The methods were applied separately to the elastic and plastic strain contributions. Table 5 summarises the values of the parameters ${\hat{\sigma }}_{f}^{\prime }$, ${\hat{\varepsilon }}_{f}^{\prime }$, ${\hat{b}}^{\prime }$, ${\hat{c}}^{\prime }$ estimated by the various statistical methods. Once the values in Table 5 are introduced in Equation (12), they provide the strain–life design curve in elastic $\varepsilon_{el,a}$, plastic $\varepsilon_{pl,a}$ and total $\varepsilon_{a}$ strain amplitude. Figure 9b compares the design curves in total strain amplitude, for a failure probability α=5% and confidence β=90%.

Figure 9b shows that, for both the elastic and plastic strain components, the method of tolerance interval (“approximate Owen’s method”) is the most conservative, since it predicts the design line that is most to the left—even though the one-side tolerance interval provides a line almost overlapped, just shifted to the right. More to the right are the design lines from the EPI and deterministic methods, although the deterministic method is even more to the right—it is, in fact, the less conservative method.

The degree of conservatism of the design curves may be appreciated by comparing the allowable strain amplitude at a prescribed fatigue life, for an assigned failure probability. The allowable strain amplitude is a value often of interest at the design stage.

The penultimate column in Table 5 lists the allowable strain amplitude $\varepsilon_{a,d}$ at ${\left(2N_{f}\right)}_{d}=2\times 10^{5}$ reversals to failure, as it is calculated from each design curve.

The last column on the right quantifies the absolute percentage reduction $e=\left|\frac{{\left(\varepsilon_{a}\right)}_{d}}{{\left(\varepsilon_{a}\right)}_{reg}}-1\right|$ of the allowable strain amplitude from regression curve, ${\left(\varepsilon_{a}\right)}_{reg}$, to the design curve, ${\left(\varepsilon_{a}\right)}_{d}$. All design curves lead to a reduction of the allowable strain compared to the regression line. The values confirm the Owen approach as the most conservative, with a reduction up to 20% is observed. Comparable, though not exactly identical, values of e would be observed for other values of ${\left(2N_{f}\right)}_{d}$, because the nonlinearity in the strain–life curve makes the reduction of strain amplitude not constant over the whole range of strains.

## 6. Conclusions

A detailed experimental analysis of the cyclic behaviour of the AISI 316L stainless steel was carried out in the present study to characterise and establish a robust procedure for durability design.

With this aim, low cycle fatigue tests at room temperature for a wide range of strain amplitudes were performed. As expected, the material showed its distinctive hardening and softening behaviour, followed by a secondary hardening. However, in contrast with other experimental observations found in the literature, the secondary hardening was present even for relatively low strain amplitudes (0.3%), with stress peaks reaching values as high as 600 MPa for larger strain amplitudes (~0.8%). Differently from what is reported in the literature, the initial yield stress of the tested material showed a much smaller value (regardless of whether the 0.2% or 0.0025% offset is considered for its assessment). Therefore, this outcome might mean that the cyclic response is somehow affected by the pre-hardening of the material, as also observed by other researchers.

In order to effectively describe the cyclic plasticity behaviour of this material within a commercial finite element code, a combined kinematic and isotropic modelling approach was used, where the kinematic part was described by means of the Chaboche model, whereas for the isotropic part a Voce model was employed. A thorough calibration of the entire set of parameters involved in the model was performed using the experimental results. This optimisation process leads to the determination of a material model capable of describing, with great accuracy, the material cyclic response for strain amplitudes up to 0.7 % and a number of cycles up to that corresponding to the end of the softening phase.

Besides the plasticity models, also the low-cycle fatigue strength was investigated. Given that the approximate strain–life curve based on the Universal Slope Equations seemed to be unsuitable to describe the strain–life relationship of the studied material, much attention was focused on the Manson–Coffin strain–life curve instead. Once the Manson–Coffin curve was obtained by regression analysis of the experimental data, several statistical methods were invoked to evaluate the design curve corresponding to a failure probability of 5% and confidence of 90%. Results emphasise that the so-called Owen approach, together with the one-side tolerance interval method, gives the most conservative strain–life curves.

Thanks to the statistical approaches employed in this paper, a more reliable design process can be achieved starting from the knowledge of the material cyclic response. This method seems to work well for uniaxially loaded materials, however, when more complex stress states are involved (i.e., multiaxial), the development of a bespoke cyclic plasticity model capable of capturing also the secondary hardening would be more suitable for durability assessment. Future works ought to address this aspect.

## Figures and Tables

**Table 1:** Chemical composition (%) of AISI 316L stainless steel tested.

%C	%Si	%Mn	%P	%S	%N	%Cr	%Mo	%Ni	%Cu	%Co
0.019	0.37	1.75	0.024	0.026	0.079	16.60	2.07	10.16	0.47	0.13

**Table 2:** Estimated material parameters used for the comparison between experimental data and simulation.

$\mathrm{Strain}\;\mathrm{Amplitude},\;\varepsilon_{a}$	Isotropic Model				Kinematic Model
	$R_{\infty ,1}$ (MPa)	$R_{\infty ,2}$ (MPa)	$b_{1}$	$b_{2}$	
0.3%	13.7	−58.9	90.00	0.8841	$C_{1}$ = 189500 MPa $\gamma_{1}$ = 2950 $C_{2}$ = 33500 MPa $\gamma_{2}$ = 350
0.4%	14.8	−51.8	46.26	0.7596	
0.5%	25.8	−49.8	29.18	0.7792	
0.6%	41.0	−48.0	16.19	0.9851	
0.7%	48.6	−42	11.37	1.280	

**Table 3:** Coefficients of the 2nd order polynomials adopted to link the isotropic model parameters for different strain amplitudes.

Parameter	$A_{1}$	$A_{2}$	$A_{3}$
$R_{\infty ,1}$ (MPa)	0.5143106	408.5609	957143.9
$b_{1}$	266.8643	−77254.43	5852143
$R_{\infty ,2}$ (MPa)	−63.42285	2054.282	128571.8
$b_{2}$	1.710213	−484.2335	60392.39

**Table 4:** Mean absolute percentage error (MAPE) of the stress peaks for each strain amplitude used for calibration and absolute percentage error (APE) for the stress peak at the end of the softening stage.

$\mathrm{Strain}\;\mathrm{Amplitude},\;\varepsilon_{a}$	Error, MAPE	Error, APE (Last Stress Peak)
0.3%	1.81%	2.19%
0.4%	0.551%	1.34%
0.5%	0.298%	0.569%
0.6%	0.233%	0.126%
0.7%	0.359%	0.0282%

**Table 5:** Estimated parameters of “median” and design strain–life curves; $\varepsilon_{a,d}$ refers to ${\left(2N_{f}\right)}_{d}=2\times 10^{5}$.

Method	K	${\left(\frac{{\hat{\sigma }}_{f}^{\prime }}{E}\right)}_{d}$	${\hat{b^{\prime }}}_{d}$	${\left({\hat{\varepsilon }}_{f}^{\prime }\right)}_{d}$	${\hat{c^{\prime }}}_{d}$	$\varepsilon_{a,d}\left(\%\right)$	e%
Regression (α=50%)	-	0.01034	−0.1748	0.05799	−0.2842	0.3031	-
USE (α=50%)	-	0.00636	−0.12	0.90604	−0.6	0.2068	31.8%
Deterministic (α=50%)	1.645	0.00890	−0.1748	0.05138	−0.2842	0.2654	12.4%
EPI (α=5% , n=8)	2.0187	0.00860	−0.1748	0.04999	−0.2842	0.2575	15.1%
1D tolerance interval (α=5%, β=90%, n=8)	2.755	0.00804	−0.1748	0.04735	−0.2842	0.2427	19.9%
1D tolerance interval Owen (α=5%, β=90%, n=8)	2.9864	0.00787	−0.1748	0.04655	−0.2842	0.2382	21.4%
${\hat{S}}_{el}$, ${\hat{S}}_{pl}$ std. deviation from regression analysis of experimental data							

**Figure A1:**
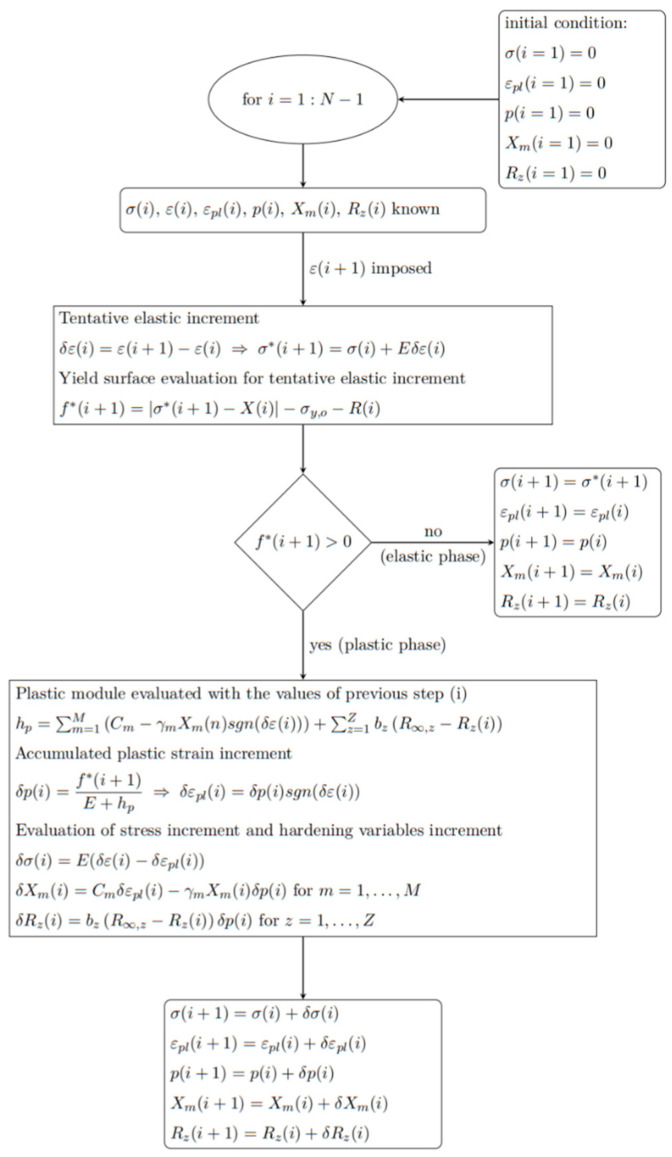
Flow chart of the algorithm used to simulate stress–strain cycles in uniaxial loading.

## Data Availability

The data presented in this study are available on request from the corresponding author. The data are not publicly available due to confidentiality reasons.

## Appendix A

This appendix describes the algorithm used to simulate the uniaxial stress–strain response obtained by a combined kinematic and isotropic model, whose parameters were calibrated on experimental data, see Table 2. The simulated cycles are compared on the experimental cycles, see for example Figure 7.

The algorithm was implemented in Matlab code and it was based on the so-called “return mapping algorithm” used in computational solid mechanics [36]. Basically, this algorithm computes the axial stress value once the axial strain history is imposed.

The strain history first needs to be discretised into a vector of *N* points ε(i), i=1, 2, …, N, which are uniformly separated by a distance equal to the strain increment δε(i)=ε(i+1)−ε(i).

Before the algorithm starts, the initial value (i=1) of some quantities is set to zero: σ(1)=0, $\varepsilon_{pl}\left(1\right)=0$, p(1)=0, $X_{m}\left(1\right)=0$, $R_{z}\left(1\right)=0$.

For each iteration step, a strain increment δε(i) is imposed and used to determine a guess value of the stress increment $\delta \sigma^{*}=E\delta \varepsilon$ in which only the elastic material behaviour is considered at first; this corresponds to the “elastic predictor phase”. The stress increment is then used in the yield criterion $f^{*}\left(i+1\right)$, see Figure A1, to check whether the material response is elastic or plastic.

If the evaluated stress with the tentative stress increment $\delta \sigma^{*}$ gives a negative value of yield criterion function $f^{*}\left(i+1\right)<0$, the material behaviour is elastic and the imposed stress increment determines the correct stress increment $\delta \sigma \left(i\right)=\delta \sigma^{*}$ which the material is subjected to. Other simulation parameters are updated accordingly. Note that the value of the yield criterion function considering the tentative stress increment is evaluated with the back stress and drag stress value in the previous iteration: $f^{*}\left(i+1\right)=\left|\sigma \left(i\right)+\delta \sigma^{*}-X\left(i\right)\right|-\sigma_{y,0}-R\left(i\right)$.

Instead, if the yield criterion function has a positive value $f^{*}\left(i+1\right)>0$, a plastic deformation takes place and the stress increment must be corrected first in order to bring it back to the yield surface (“plastic corrector phase”). By means of this correction, and after calculating the plastic modulus $h_{p}\left(i\right)=\delta \sigma /\delta \varepsilon_{pl}$, the algorithm can determine the accumulated plastic strain increment δp(i) that is used to calculate the plastic strain increment $\delta \varepsilon_{pl}$. Again, the simulation parameters are updated accordingly, see Figure A1.

The algorithm ends when the last point in vector ε(i) has been analysed.

One of the main problems for this algorithm is that it does not have any iterative scheme to guarantee numerical convergence at each iteration, i.e., at each increment of imposed strain. More precisely, the algorithm makes use of an explicit integration scheme by which, at each iteration, the plastic modulus is computed by using the values computed in the preceding iteration, although the plastic modulus is actually a function of the parameter values at the current step. Nevertheless, convergence is achieved—and the algorithm then provides accurate results—if small increments of strain δε are chosen at each iteration.

Despite this minor drawback, this type of algorithm is highly recommended as it is computationally inexpensive and thus allows for a fast check of the parameters estimated from experiments.
